# Structural Variants May Be a Source of Missing Heritability in sALS

**DOI:** 10.3389/fnins.2020.00047

**Published:** 2020-01-31

**Authors:** Frances Theunissen, Loren L. Flynn, Ryan S. Anderton, Frank Mastaglia, Julia Pytte, Leanne Jiang, Stuart Hodgetts, Daniel K. Burns, Ann Saunders, Sue Fletcher, Steve D. Wilton, Patrick Anthony Akkari

**Affiliations:** ^1^Perron Institute for Neurological and Translational Science, Nedlands, WA, Australia; ^2^School of Human Sciences, University of Western Australia, Nedlands, WA, Australia; ^3^Centre for Neuromuscular and Neurological Disorders, University of Western Australia, Nedlands, WA, Australia; ^4^Centre for Molecular Medicine and Innovative Therapeutics, Murdoch University, Perth, WA, Australia; ^5^School of Health Sciences, Institute for Health Research, University of Notre Dame Australia, Fremantle, WA, Australia; ^6^School of Biological Sciences, University of Western Australia, Nedlands, WA, Australia; ^7^Zinfandel Pharmaceuticals, Chapel Hill, NC, United States

**Keywords:** amyotrophic lateral sclerosis, structural variant, genetic marker, missing heritability, clinical trial stratification

## Abstract

The underlying genetic and molecular mechanisms that drive amyotrophic lateral sclerosis (ALS) remain poorly understood. Structural variants within the genome can play a significant role in neurodegenerative disease risk, such as the repeat expansion in *C9orf72* and the tri-nucleotide repeat in *ATXN2*, both of which are associated with familial and sporadic ALS. Many such structural variants reside in uncharacterized regions of the human genome, and have been under studied. Therefore, characterization of structural variants located in and around genes associated with ALS could provide insight into disease pathogenesis, and lead to the discovery of highly informative genetic tools for stratification in clinical trials. Such genomic variants may provide a deeper understanding of how gene expression can affect disease etiology, disease severity and trajectory, patient response to treatment, and may hold the key to understanding the genetics of sporadic ALS. This article outlines the current understanding of amyotrophic lateral sclerosis genetics and how structural variations may underpin some of the missing heritability of this disease.

## Amyotrophic Lateral Sclerosis; Clinical Phenotypes and Heritability

Amyotrophic lateral sclerosis (ALS) is a devastating progressive neurodegenerative disorder characterized by the loss of lower and upper motor neurons, resulting in paralysis of the limb, bulbar and respiratory muscles, and is typically fatal within 3–5 years from disease onset (Robberecht and Philips, 2013). The disease incidence is ∼2 per 100,000 population and it is projected that rates of ALS will increase from ∼222,000 worldwide in 2015 to ∼376,000 in 2040 (Arthur et al., 2016). In addition to the growing financial burden of this disease, there is a significant burden on the patients’ families and carers. As such, it is critical to improve our understanding of how genetic mechanisms may contribute to the pathogenesis of this devastating disease. Investigation of short structural variations (SVs) in known ALS genes has multiple potential objectives, and may help to uncover some of the missing heritability in sporadic ALS. Characterization of short SVs may inform the discovery of novel disease mechanisms and therapeutic targets, and be useful for stratification of patient subgroups in clinical trials.

ALS is a heterogeneous disease, with variable clinical presentation between patients, and is characterized by progressive motor deficits that evolve over weeks or months, eventually affecting most voluntary muscles in the body (Talman et al., 2016). The heterogeneity of clinical presentation and diverse rates of progression make the disease challenging to diagnose, and there is currently no definitive diagnostic test. As a result, it is usually characterized on the basis of the site and pattern of onset, and the degree of involvement of upper and lower motor neurons, and can be classified into the following categories: (i) progressive bulbar palsy (difficulty swallowing and speech disturbances); (ii) limb-onset ALS; (iii) progressive muscular atrophy (involving only lower motor neurons); and (iv) upper motor neuron predominant ALS (Kinsley and Siddique, 2015). Most commonly, individuals will present with asymmetrical focal weakness of the extremities (poor hand grip, foot drop, stumbling) or bulbar characteristics (dysarthria and dysphagia). Other typical symptoms include muscle fasciculation, cramps and hyperreflexia in regions of atrophy, without accompanying sensory disturbances (Kinsley and Siddique, 2015). Importantly, the different clinical phenotypes exhibit differing rates of progression, with the bulbar-onset form having the most rapid progression and shortest survival time (Okamoto et al., 1993). In addition, there is considerable variability between cases within the same diagnostic category. Given the degree of heterogeneity, it remains to be determined whether the different clinical phenotypes all represent variations of the same disease, or whether there is also heterogeneity in the underlying genetic and molecular disease determinants. At present, there is a lack of specific genetic or other biomarkers for the different disease subtypes, or indicators of disease trajectory in well-documented patient cohorts.

Approximately 10% of ALS cases are classed as familial (fALS), while the remainder, with no prior family involvement, are classified as having the sporadic form of the disease (sALS) (Renton et al., 2014). Since the landmark discovery of the first mutation in the superoxide dismutase 1 gene *(SOD1)* in fALS in the early 1990s (Rosen et al., 1993), there has since been significant progress in understanding of the genetics of the familial disease, with approximately 70% of the genetic mutations that contribute to fALS having been identified (Cook and Petrucelli, 2019). However the genetic underpinnings of sporadic ALS (sALS) remain a formidable challenge (Al-Chalabi et al., 2012; Renton et al., 2014). Comparatively, few mutations have been described for sALS, despite the application of high throughput genetic analysis methods (Nguyen et al., 2018). These methodologies have failed to identify disease-associated genetic variations in the majority of sALS patients, highlighting the complexity and genetic heterogeneity contributing to this disease phenotype. Approximately 10% of sALS cases can be explained by mutations in 25 known ALS-linked genes, with the remaining 90% of cases as yet having an undetermined genetic contributor (Andersen and Al-Chalabi, 2011; Renton et al., 2014; Dharmadasa et al., 2017). At a glance these data may imply that the genetic contributions to sALS are minor, however, heritability estimates and twin studies suggest a genetic contribution of up to 65% (Al-Chalabi et al., 2010; Al-Chalabi and Visscher, 2014). It is therefore likely that additional genetic contributors to sALS risk remain to be identified. The situation may be analogous to that for Alzheimer’s disease where the *APOE* ε4 (Apolipoprotein E) risk allele has a frequency of only 14% and does not fully explain the Alzheimer’s disease age-of-onset risk (Corder et al., 1993; Roses et al., 2016). However, after discovery of the structural variant (rs10524523) in the neighboring *TOMM40* (Translocase of outer mitochondrial membrane 40) gene, age of onset risk could now be assessed for >90% of the at risk population (Roses et al., 2010).

Currently there are only two therapeutics available for the treatment of ALS, Riluzole (approved in Australia, United States, and Europe) and Edaravone (approved in Japan, South Korea, and United States) (Rothstein, 2017) that impact excitotoxicity (Doble, 1996) or anti-oxidant pathways (Rothstein, 2017), respectively and may slow disease progression for a relatively short period of time. However, these drugs are only effective in some patients and there is currently no way to determine those most likely to respond to the drugs. For the patients that do show a response, life is only prolonged by approximately 3 months. Further understanding of ALS pathogenesis should inform the development of more effective therapies and help identify patients likely to respond to specific treatments.

## Genetic Characterization and Genome-Wide Association Studies

Genome wide association studies analysing single nucleotide polymorphisms (SNP) and whole exome sequencing studies have provided a wealth of information relating to common variants associated with a range of diseases. Despite this, such approaches have generally identified genes that are either inherited in fALS, those that are weakly associated with sALS, or mutations associated with rare forms of the disease (Renton et al., 2014). Some of the ALS genes identified by these techniques include *UNC13A, C9orf72, DPP6, ELP3*, *KIFAP3, TBK1, CHCHD10, TUBA4A, CCNF, MATR3, NEK1, C21orf2, ANXA11*, and *TIA1* (see Table 1; Andersen and Al-Chalabi, 2011; Nguyen et al., 2018). A major issue with utilizing these technologies is that by testing thousands of SNPs across the genome, low effect size associations are generated for numerous gene regions, inevitably leading to very high thresholds of significance for potentially weak genetic effects (Roses, 2016). This can lead to false positive associations or a lack of reproducibility between different populations that have rarely translated into tools for clinical trial patient stratifications, with the exception of *UNC13A* (see below). The amount of information these techniques can provide regarding complex disease and the functional outcomes of SNPs are limited. Particularly, these methods have not been able to account for the variation in age-of-onset and progression in ALS patients (Al-Chalabi and Hardiman, 2013), and fail to explain the missing heritability of the disease (Al-Chalabi et al., 2017; Mejzini et al., 2019). Whole genome sequencing can examine the entirety of the genome to better capture larger scale variations, as opposed to single nucleotide changes, however, these methods come with their own technical limitations; high throughput short-read sequencing technologies are unable to accurately capture these regions due to amplification stutter, and misaligning of the short-read sequences, often misrepresenting their true variability (Cameron et al., 2019). Recently, it was demonstrated that variable areas of the genome can camouflage each other, particularly where short-read DNA sequences map equally well to different loci, resulting in poor SV characterization (Ebbert et al., 2019). To date, these techniques have only accounted for the genetic cause of approximately 10% of sALS cases (Cook and Petrucelli, 2019). Therefore, it is essential to rethink the approach and acknowledge the limitations of these technologies when interrogating the genome. Specifically, there are vast regions of genetic variability yet to be uncovered in non-coding regions, which might have significant implications in the context of complex disease.

**TABLE 1:** This table lists published genes that have been associated with ALS and highlights the discovery method for each gene as well as the gene function.

ALS genes	Discovery method	Gene function	References	Number of predicted short SVs
*C9orf72*	GWAS	RNA metabolism	Morita et al., 2006	31
*TARDP*	Candidate gene linkage	RNA metabolism	Gitcho et al., 2008	63
*FUS*	Candidate gene linkage	RNA metabolism	Vance et al., 2009	45
*MATR3*	WES	RNA metabolism	Johnson et al., 2014	60
*TIA1*	WES	RNA metabolism	Cirulli et al., 2015	83
*HNRNPA1*	Linkage WES	RNA metabolism	Kim et al., 2013	23
*HNRNPA2/B1*	Linkage WES	RNA metabolism	Kim et al., 2013	24
*EWSR1*	Candidate gene	RNA metabolism	Couthouis et al., 2012	66
*TAF15*	Candidate gene	RNA metabolism	Ticozzi et al., 2011	35
*ANG*	Candidate gene	RNA metabolism	Greenway et al., 2006	25
*SMN1*	Candidate gene	Interaction with RNA binding proteins	Corcia et al., 2002	68
*ELP3*	GWAS	Transcript elongation	Simpson et al., 2008	62
*SETX*	Linkage	DNA/RNA processing	Chen et al., 2004	101
*SPG11*	Linkage	DNA damage	Orlacchio et al., 2010	110
*APEX1*	Candidate gene	Endonuclease	Greenway et al., 2004	13
*UBQLN2*	Candidate gene linkage	Protein quality control	Deng et al., 2011	9
*VCP*	Candidate gene	Protein quality control	Johnson et al., 2010	37
*OPTN*	Homozygosity mapping	Protein quality control	Maruyama et al., 2010	54
*VAPB*	Linkage	Protein quality control	Nishimura et al., 2004	64
*TBK1*	WES	Protein quality control	Freischmidt et al., 2015	59
*SQSTM1*	Candidate gene	Protein quality control	Fecto et al., 2011	38
*CCNF*	Genome wide linkage	Protein quality control	Williams et al., 2016	48
*PFN1*	Linkage WES	Cytoskeletal and trafficking	Wu et al., 2012	21
*TUBA4A*	WES	Cytoskeletal and trafficking	Smith et al., 2014	24
*KIF5A*	GWAS	Cytoskeletal and trafficking	Nicolas et al., 2018	52
*ANXA11*	WES	Cytoskeletal and trafficking	Smith et al., 2017	41
*NEFH*	Candidate gene	Cytoskeletal and trafficking	Figlewicz et al., 1993	43
*DCTN1*	Candidate gene	Cytoskeletal and trafficking	Münch et al., 2004	18
*PRPH*	Candidate gene	Cytoskeletal protein	Leung et al., 2004	50
*FIG4*	Candidate gene	Cytoskeletal organization and vesicle trafficking	Chow et al., 2009	66
*CFAP410*	GWAS	Cytoskeletal and DNA damage response	Van Rheenen et al., 2016	15
*KIFAP3*	GWAS	Kinesin associated protein	Landers et al., 2009	55
*ALS2*	Linkage	Endosomal dynamics	Hadano et al., 2001	69
*SIGMAR1*	Homozygosity mapping	Endoplasmic reticulum chaperone	Al-Saif et al., 2011	21
*SOD1*	Linkage	Mitochondrial dysfunction and oxidative stress	Rosen et al., 1993	30
*CHCHD10*	Candidate gene	Mitochondrial dysfunction and oxidative stress	Bannwarth et al., 2014	32
*NEK1*	WES	Mitochondrial dysfunction and oxidative stress	Kenna et al., 2016	99
*ATXN2*	Candidate gene	Endocytosis, cell survival	Elden et al., 2010	186
*GRN*	Candidate gene	Cell growth regulator	Schymick et al., 2007	34
*UNC13A*	GWAS	Neurotransmitter release	Van Es et al., 2009	103
*PLCD1*	GWAS	Signal transduction	Staats et al., 2013	21
*CHMP2B*	Candidate gene	Recycling of cell receptors	Parkinson et al., 2006	22
*ITPR2*	GWAS	Receptor	Van Es et al., 2007	204
*ARHGEF28*	Candidate gene	Nucleotide exchange factor	Droppelmann et al., 2013	138
*DAO*	Candidate gene	Potential detoxifying agent	Mitchell et al., 2010	52
*DPP6*	GWAS	Modifies calcium gated channels	Van Es et al., 2008	235
*VEGFA*	Candidate gene	Angiogenesis, migration of endothelial cells	Lambrechts et al., 2003	15
*HFE*	Candidate gene	Iron absorption	Wang et al., 2004	31
*PON1*	Candidate gene	Organophosphate hydrolysis	Slowik et al., 2006	24

**Using the method described by Saul et al. (2016) we have predicted the number of short structural variants in each gene that may warrant further investigation. This does not include insertion/deletions or SNPs.**

## Structural Variants

Structural variants (SVs) are defined as insertions, deletions, inversions and microsatellites that can be repeated hundreds of times. SVs predominantly occur in non-coding regions of the genome and often do not change the composition of the mature protein (Roses et al., 2016). Despite this, changes in the size and composition of SVs can have a significant impact on the regulatory elements that modulate gene expression (Chiang et al., 2017). Therefore, SVs can potentially provide a deeper understanding of how gene expression in complex genetic disease can affect disease etiology, duration, progression and patient outcomes (Feuk et al., 2006). SVs have been implicated in many complex diseases including retinitis pigmentosa (*MSR1*) (Rose et al., 2016), Alzheimer’s (*TOMM40*) (Lyall et al., 2013), frontotemporal dementia (*C9orf72*) (DeJesus-Hernandez et al., 2011; Renton et al., 2011), and other neurodegenerative diseases (Beck et al., 2013).

**FIGURE 1:**
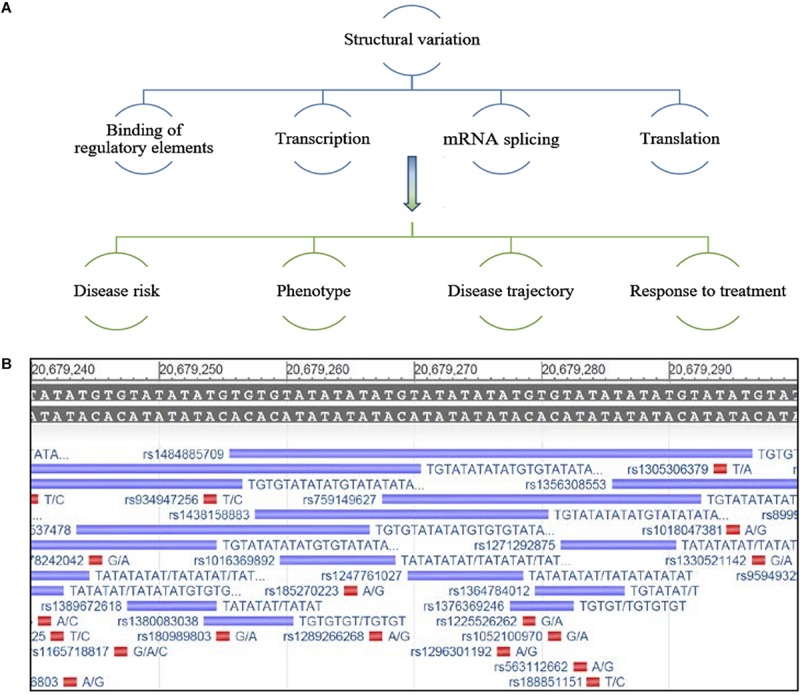
**(A)** Schematic representing potential regulation of gene expression by structural variants and possible effects on disease characteristics. **(B)** Example of an under-characterized gene from the NCBI database GRch38.p13 primary assembly, a region that has been repeatedly mapped but lacks consensus around the nature of this repeat sequence and its true variability. The reference sequence is located at the top of the image, the purple bars depict a sequencing entry with variable size and its associated rs number and red bars represent a SNP entry (Gene [Internet]. Bethesda (MD): National Library of Medicine (US), National Center for Biotechnology Information; 2004 – [cited 2019 September 24]. Available from: https://www.ncbi.nlm.nih.gov/gene/).

The ability of SVs to alter gene expression is likely dependent on their location within and around the gene or intergenic region, with their effects occurring via several mechanisms including, influencing the binding of regulatory elements that determine transcription, mRNA splicing and processing, genome folding and higher order structure, and translation (Roses et al., 2016). This may differentiate mechanisms of disease pathogenesis, including risk of disease, risk for a specific phenotype, symptom presentation, disease course and response to treatment, between individuals (Figure 1A). Due to the variable nature, as well as the repeat structure of SVs, many remain poorly characterized by analysis platforms such as next generation sequencing (Cameron et al., 2019; Ebbert et al., 2019).

Historically, research on SVs has focused on genomic elements that are larger in size (>1 kb) and much easier to capture by high throughput techniques, such as copy number variations, transposable elements, larger insertion/deletions, translocations, and duplications (Sebat et al., 2004; Feuk et al., 2006; Huang et al., 2010; Alkan et al., 2011; Sudmant et al., 2015); yet other small variable regions of the genome remain under characterized and are more difficult to capture with short-read sequencing techniques (Figure 1B; Chaisson et al., 2019; Ebbert et al., 2019). Recently, a comprehensive review highlighted the strengths and weakness of various sequencing methods and bioinformatics tools used for SV calling, and the difficulties in capturing smaller novel variations (Mahmoud et al., 2019). In addition, comparing the accuracy of SV characterization between methodologies when different *in silico* data sets are often utilized remains a challenge (Mahmoud et al., 2019). Furthermore, how these methods translate and generalize to patient samples is unclear, making it critical to establish standard procedures and bench-marks for the interpretation of SV data. There is a growing need to utilize SV information in the clinical setting to inform variation in patient phenotypes. Therefore we and others believe, important genes that may be critical to understanding the variation between patients that cannot be fully explained by SNPs or other mutations, should be reinvestigated for short SVs. Unrecognized or under characterized SVs could influence the expression of these genes, thereby contributing to the risk of ALS.

Recent studies investigating the transcriptome of the spinal cord anterior horn have identified significantly different RNA profiles between ALS patients and controls, for a multitude of gene pathways (D’Erchia et al., 2017). Since SVs can exhibit a range of regulatory effects that can impact levels of gene expression and potentially the phenotype, it is essential that these regions are also properly characterized. For example, a microsatellite repeat element in the promotor region of *PRPF31* (precursor mRNA-processing factor 31) results in some mutation carriers developing retinitis pigmentosa, whilst others remain asymptomatic (Rose et al., 2016). The length of this SV was shown to impact the penetrance of the mutation by suppressing transcription of this region by 50–115-fold, resulting in haploinsufficiency (Rose et al., 2016). Such disease mechanisms also warrant investigation in ALS.

### STRUCTURAL VARIANTS IN ALS: *C9orf72* AND *ATXN2* VARIATION

An example of an SV that results in ALS pathogenesis is the repeat expansion in the *C9orf72* gene. The protein encoded by this gene is thought to play a role in endosomal membrane trafficking and autophagy (Farg et al., 2014). The SV region of *C9orf72* is a hexanucleotide repeat located in intron 1, GGGGCC that is usually repeated up to 30 times in healthy individuals. Expansion of this repeat to hundreds or thousands of repeated segments is a recognized cause of fALS, frontotemporal dementia, and occasionally also sALS (Mis et al., 2017). The DNA encoding this repeat is transcribed bi-directionally, resulting in nuclear RNA inclusions, and is thought to promote gain of function toxicity (Ly and Miller, 2018; Staats et al., 2019). Other potential mechanisms include *C9orf72* loss of function (Shi et al., 2018; Staats et al., 2019), or proteotoxicity (Gitler and Tsuiji, 2016). In particular, patients with expanded repeats have a more severe phenotype, are predominantly bulbar onset, exhibit an earlier age of disease onset, cognitive and behavioral impairment and reduced survival (Byrne et al., 2012; Cooper-Knock et al., 2014). In addition, microsatellite repeats are subjected to an unconventional mechanism called repeat associated non-ATG (RAN) translation (Zu et al., 2011), whereby proteins can be coded by the additional two reading frames (GCA and AGC) resulting in up to six dipeptide proteins (Cleary and Ranum, 2014, 2017). The accumulation of these dipeptide proteins is seen in the central nervous system of individuals with ALS and contributes to a multitude of mechanisms that can induce protein-mediated toxicity (Balendra and Isaacs, 2018). One of the current strategies employed in the development of therapeutics for *C9orf72* is to target the SV region with antisense oligonucleotides to induce transcript degradation by RNase H enzymatic cleavage, preventing the build-up of toxic *C9orf72* transcript and protein. Other strategies focus on modulating the expression of transcription factors specifically involved in transcribing expanded repeats (Ly and Miller, 2018). Recently, it was shown that small ribosomal subunit protein (RPS25) plays a direct role in RAN translation, and decreasing its levels through RNA interface mediated reduction prolonged the lifespan of *Drosophila* with the expanded repeat (Yamada et al., 2019). In addition, antisense oligonucleotide targeting of RPS25 enhanced the survival of *C9orf72* derived motor neurons reducing poly-GR and poly-PR foci (Yamada et al., 2019). The recent identification of a RAN translation regulator is a big step forward in demonstrating the functional implications of SV repeats in disease, and indicates antisense oligonucleotide or small molecules could be a viable therapeutic option for targeting RPS25 for patients with *C9orf72* (Hutten and Dormann, 2019). However, further research is needed to understand the regulation of expanded repeats and determine the relative contribution of repeat RNA and dipeptide repeat proteins to patient phenotype and cellular toxicity that promotes neurodegeneration in ALS.

Another SV contributing to ALS pathogenesis occurs in the gene encoding ataxin-2 (*ATXN2)*. *ATXN2* contains a CAG repeat, initially found to be associated with a class of neuromuscular and neurological disorders, known as polyglutamine disorders, caused by the expansion of the microsatellite repeat within the coding sequence (Al-Chalabi et al., 2012; Lattante et al., 2014). The ATXN2 protein is involved in endocytosis and modulates mTOR signals, critical to maintaining cell growth and survival, thereby modifying translation and mitochondrial function (Carmo-Silva et al., 2017). The N-terminal of this protein contains a polyglutamine tract that generally consists of 14–31 residues that when expanded, can carry up to 200 residues in the pathogenic state (Sproviero et al., 2017). Long expanded repeats were initially found to cause spinocerebellar ataxia 2 and subsequently, intermediate length repeats were shown to increase the risk of developing ALS (Daoud et al., 2011; Van Damme et al., 2011), with one study demonstrating that ALS risk increases exponentially with repeat length until the cut-off for developing spinocerebellar ataxia is reached (Sproviero et al., 2017). The polyglutamine disease causing mechanism differs between conditions and may include a loss of function resulting from hyper-methylation (Jin and Warren, 2000), a toxic gain of function through RAN translation (Scoles et al., 2015), protein misfolding and aggregation (Kayatekin et al., 2014), and in ALS, increasing TDP-43 toxicity (Elden et al., 2010). Longer repeats were expected to result in increased toxic TDP-43 build-up, resulting in increased risk of disease, an earlier age of onset and faster progression, however, this is not necessarily seen in patients (Al-Chalabi and Hardiman, 2013). The possibility of oligogenic inheritance is the likely explanation, where multiple risk factors from polymorphic structural variants are required to act together over time, with environmental stressors, to cause the development of ALS (Al-Chalabi et al., 2014), accounting for the particular variability seen in age-of-onset and disease progression. A better understanding of these variable regions of the genome and how they might work together to cumulatively increase disease risk, resulting in motor neuron dysfunction and susceptibility to neuronal degeneration, is imperative.

## Therapeutic Development Challenges

Over the past 20 years, more than 50 controlled trials of putative ALS therapeutics, testing 60 molecules have failed to demonstrate clinical efficacy (Petrov et al., 2017). Transgenic *SOD1* mice have been used for the majority of ALS pre-clinical drug development studies; however, these models do not translate well to human disease (Mitsumoto et al., 2014). Alternative approaches need to be used to evaluate the potential efficacy of compounds. Identification and utilization of genetic markers, such as SVs informative for ALS, could be incorporated into clinical trial design to reduce the participant heterogeneity (Van Eijk et al., 2019).

Poor understanding of the pathogenic mechanisms of ALS neuro-degeneration remains a barrier to the development of novel therapeutic approaches. Moreover, there are few biomarkers that allow patient stratification according to disease mechanism (Agah et al., 2018; Khalil et al., 2018; Mitsumoto and Saito, 2018; Vejux et al., 2018). As a result, efficacy can only be evaluated by clinical measures during clinical trials (Mitsumoto et al., 2014; Mitsumoto and Saito, 2018). Clearly, there is a critical unmet need to establish well-characterized molecular biomarkers that can be used as therapeutic targets, or to inform on the validity of certain treatment approaches. As ALS is a complex, heterogenous disorder with a varied age of onset and expression, it is likely that no single therapeutic will be effective for all patients. Therefore, we must develop strategies to identify patient subgroups and develop compounds to address the specific molecular defect.

Establishing molecular targets and markers for ALS could lead to improved patient stratification for clinical trials, to enable treatment effects to be identified within specific patient sub-groups. An example of the success of this approach is provided by clinical research with lithium carbonate in ALS patients. After a pilot study demonstrated attenuation of disease progression in a small number of ALS patients treated with lithium carbonate (Fornai et al., 2008), a number of follow up clinical studies have failed to replicate the finding (Aggarwal et al., 2010; Chiò et al., 2010; Miller et al., 2011; Verstraete et al., 2012; UKMND-LiCALS Study Group, 2013). In a meta-analysis of three trials that failed to show a significant effect of lithium treatment in ALS, Van Eijk et al. (2017) retrospectively demonstrated that lithium-treated patients who carried the *UNC13A* (C/C) genotype had a slower disease trajectory and showed a 70% improvement in 12 month survival, whilst carriers of the same genotype receiving no treatment had significantly reduced survival trajectories (Van Eijk et al., 2017). This survival benefit was only evident once the patients were stratified by their genotype, as the heterogeneous trial cohort originally masked the identification of any therapeutic benefit for a subgroup. In a more recent report Van Eijk et al. (2019) demonstrated that different genotypes including the repeat expansion *C9orf72* can interact with both primary and secondary endpoints of clinical trials. Interestingly, in this report *C9orf72* carriers did not have reduced survival, however, they did exhibit an accelerated monthly decline measured by ALSFRS compared to non-carriers. No pharmacogenetic interactions were demonstrated in the valproic acid trial, however, there was a pharmacognetic interaction between creatine treatment and the A allele of *MOBP*, whilst a dose response was observed for the C allele of *UNC13A* improving survival outcomes (Van Eijk et al., 2019). This highlights the importance of taking genetic information into account in clinical trials to enrich trial populations for potential responders. Identifying new genetic variations that may explain changes in gene expression in sALS patients will therefore be extremely useful to help inform both primary and secondary end points in clinical trial, and may improve the likelihood of clinical trial success.

## Concluding Remarks

The methodologies currently used to elucidate ALS pathogenesis and inform drug development have not delivered effective therapeutic strategies to date. In our view, continuing to perform further genome wide association studies is unlikely to provide the breakthroughs that are urgently needed. Genome wide studies can sometimes identify biochemical pathways involved in disease and indicate genes associated with fALS and sALS, however, in-depth characterization of these implicated regions may identify SVs that influence ALS susceptibility. Limitations of GWAS and even whole genome sequencing in identifying ALS risk must be recognized, since allele frequencies of variants or SNPs may not differ between patient cohorts and controls in these complex diseases. With increasing likelihood that SVs do indeed contribute to ALS risk, future investigations will need to incorporate SVs into genetic studies. It is possible that a combination of variants occurring frequently throughout healthy populations will collectively contribute to the vulnerability of motor neurons, and that this could be further exacerbated by both physiological and environmental insults. Particular SVs may better account for the variability in phenotypes and progression across ALS patient cohorts, and could be critical targets that can inform drug development. As our current molecular understanding of ALS has proven largely ineffective in easing the burden of ALS, clinical trials are likely to continue to fail if analyses are restricted to conventional strategies and platforms.

*In silico* investigations by our laboratory group reveal that there are a number of under-characterized genomic regions in ALS genes. Genetic data-bases including National Center for Biotechnology Information^1^, Ensembl genome browser 97^2^ and University of California Santa Cruz genome browser^3^ have multiple sequence entries logged for genomic loci under different RS numbers. Sequencing technologies, whilst sometimes precise, have limited accuracy (Roses, 2016), as they only provide the location but not the specifics of each variant in individual patients. These poorly characterized regions of the genome could therefore contribute to the missing heritability of ALS. In most cases, the variation in allele length and allele frequency remains unclear, ultimately leading to the question, “how significant a role do SVs play in complex diseases, such as ALS?” We have focused on the development of accurate assays to genotype SVs. For example, investigation of the *SOD1* region has led to the characterization of SV1, a variant located within the 3′ untranslated region of *SCAF4*, a downstream gene that was previously overlooked. The function of *SCAF4* has recently been elucidated, with the protein being necessary for accurate termination of transcription by ensuring correct polyadenylation site selection (Gregersen et al., 2019). This SV appears to influence *SOD1* expression, possibly through a toxic gain of function, and could more broadly influence ALS pathogenesis (Roses Allen, 2019). Continued investigations into the function of SV1 are presently underway in our laboratory. In addition, other variants appear to stratify sALS patients on the basis of survival and may in fact act as modifiers of gene expression (unpublished data). If this data is confirmed, it will not only indicate novel mechanisms contributing to ALS, but also allow patient stratification for enrichment of ALS clinical trials. Although SVs may not always be a viable drug target, they may indicate pathways that can be targeted to inform drug development. It is our belief that we need to re-assess these regions of the genome in order to identify some of the missing heritability of ALS and explain the phenotypic variability seen across this disease.

## Author Contributions

FT, RA, and PA contributed to the content addressed in the perspective. FT wrote the first draft of the manuscript. All authors contributed to the manuscript revision, read, and approved the submitted version.

## Conflict of Interest

DB and AS are employed by the company Zinfandel Pharmaceuticals. The patent for “Methods for detecting structural variants in neurodegenerative disease,” invented by Allen D. Roses, is now owned by AS. The remaining authors declare that the research was conducted in the absence of any commercial or financial relationships that could be construed as a potential conflict of interest.

## Footnotes

1 https://www.ncbi.nlm.nih.gov/gene/

2 http://asia.ensembl.org/index.html

3 https://genome.ucsc.edu/cgi-bin/hgGateway
